# Glioma Stemlike Cells Enhance the Killing of Glioma Differentiated Cells by Cytotoxic Lymphocytes

**DOI:** 10.1371/journal.pone.0153433

**Published:** 2016-04-13

**Authors:** Esen Yonca Bassoy, Valentina Chiusolo, Guillaume Jacquemin, Cristina Riccadonna, Paul R. Walker, Denis Martinvalet

**Affiliations:** 1 Department of Cell Physiology and Metabolism University of Geneva, 1211, Geneva, Switzerland; 2 Geneva University Hospitals and University of Geneva, Centre of Oncology, 1211, Geneva, Switzerland; University Hospital of Navarra, SPAIN

## Abstract

Glioblastoma multiforme, the most aggressive primary brain tumor, is maintained by a subpopulation of glioma cells with self-renewal properties that are able to recapitulate the entire tumor even after surgical resection or chemo-radiotherapy. This typifies the vast heterogeneity of this tumor with the two extremes represented on one end by the glioma stemlike cells (GSC) and on the other by the glioma differentiated cells (GDC). Interestingly, GSC are more sensitive to immune effector cells than the GDC counterpart. However, how GSC impact on the killing on the GDC and *vice versa* is not clear. Using a newly developed cytotoxicity assay allowing to simultaneously monitor cytotoxic lymphocytes-mediated killing of GSC and GDC, we found that although GSC were always better killed and that their presence enhanced the killing of GDC. In contrast, an excess of GDC had a mild protective effect on the killing of GSC, depending on the CTL type. Overall, our results suggest that during combination therapy, immunotherapy would be the most effective after prior treatment with conventional therapies.

## Introduction

Glioblastoma multiforme (GBM), the highly heterogeneous and the most frequent and lethal primary brain tumor [1], is refractory to conventional therapy combining surgical resection, radiotherapy and chemotherapy [2]. Patients diagnosed with this difficult to cure disease only have 14.6 months of median survival [1, 3, 4]. Genetic, hierarchical and functional diversities, epigenetics, and tumor microenvironment are all contributing factors of the intra- and inter-tumor complexity [5–11]. The different types of tumor cells are involved in interaction with neighboring stromal and cancer cells, or with the immune infiltrate constituting the microanatomy of the tumor. The integration of these different parameters will most likely dictate the tumor responsiveness to therapeutic interventions [12, 13]. In the case of GBM, this vast heterogeneity is also exemplified by the presence of a subpopulation of cancer initiating cells with stemlike potential called glioma stemlike cells (GSC). GSC and their glioma differentiated cell (GDC) counterpart would be the two extremes of the spectrum of variability comprising the highly heterogeneous GBM mass *in vivo* [10, 11, 14, 15]. The proportion of this cancer stemlike cell population in the tumor mass is proposed to be an indication of the tumor aggressivity and of a poor prognosis [13, 16]. Therefore, therapeutic interventions aiming at eliminating GSC could have the promise of durable treatment response. Nevertheless, among other features, due to their quiescence and robust DNA repair machinery, conventional therapies are poorly efficacious against GSC [13, 17–19]. Conceptually, harnessing the power of the endogenous immune response against GSC is a very attractive alternative to eradicate GSC during immunotherapy. Indeed, both GDC and GSC can be efficiently targeted by cytotoxic immune effector cells [20–26]. These findings, together with identification of multiple glioma antigens [27], have led to the development of vaccines eliciting coordinated multi-epitope T cell-mediated immunity, T helper functions, and immunologic memory [28]. Interestingly, GSC were observed to be more sensitive than GDC to cytotoxic T lymphocytes (CTL) and natural killer (NK) cells [24]. Because of the intricate interconnexion and interaction between the different cell types constituting the tumor, cytotoxic immune cells are likely to sometimes encounter GSC and GDC simultaneously depending of the microanatomy and hierarchical organization of the tumor. Nevertheless, the influence of GSC on GDC killing and *vice versa* is not clear. To address this question, we have used a novel two-color calcein release assay that allows the monitoring of the cytotoxicity toward two types of target cells simultaneously. We first confirmed that both human and mouse glioma stemlike cells are more sensitive to NK cells and CTL. We found that regardless of the GSC: GDC ratio, GSC are better killed than GDC by CTL. A protective effect of an excess of GDC on the cytotoxicity toward GSC was observed; it was modest and depended on the maturity, the potency and the type of CTL. Interestingly, the presence of GSC enhanced the killing of GDC. Taken together, our results show that because of the influence of the different cancer cell types on CTL killing efficiency, immunotherapy would be most effective after treatment of the tumor mass with conventional therapies.

## Material and Methods

### Cell culture and Reagents

U251 and GL261 cells were grown in Dulbecco's modified Eagle's medium (DMEM) (Gibco) with 10% fetal bovine serum (FBS) (Gibco) supplemented with 100 U/ml penicillin G (Sigma-Aldrich), 100 μg/ml streptomycin sulfate (Sigma-Aldrich), 6mM hepes (Applichem), 1.6 mM L-glutamine (Sigma-Aldrich), 50 μM β-mercaptoethanol (Biorad). U251 and GL261 harvested using accutase (Invitrogen) according to manufacturer’s procedure. To obtained hNS, U251 were dedifferentiated and maintained in neurosphere medium (DMEM/F12) medium supplemented with, 100 U/ml penicillin G, 100 μg/ml streptomycin sulfate, 6mM hepes, 1.6 mM L-glutamine, 50 μM β-mercaptoethanol, B-27 supplement (Invitrogen), 20 ng/mL recombinant human basic fibroblast growth factor (basic-FGF) (Invitrogen,) and 20 ng/ml recombinant human epidermal growth factor (EGF) (Invitrogen) and 50 U/ml heparin (Sigma). To obtained mNS, GL261 cells were dedifferentiated in the same medium using mouse basic FGF and mouse EGF. For each passage, spheres were dissociated with accutase (Invitrogen) and passaged into fresh media. YT-Indy NK cells were grown in RPMI 1640 supplemented with 10% fetal calf serum, 100 U/ml penicillin G, 100 μg/ml streptomycin sulfate 6 mM hepes free acid, 1.6 mM L-glutamine, and 50 μM β-mercaptoethanol.

### Mice and CTL isolation

PMEL and OT-1 mice (Charles River) were bred in house and used between 6–12 weeks of age according to approved animal protocols by the Directorate General for Health (DGS) of the Department of Employment, Social Affairs, and Health (DEAS). This study was carried out in strict accordance with the Swiss Regulation and the protocol was approved by the Cantonal Commission for Animal Experiment of the Directorate General for Health of the Department of Employment, Social Affairs, and Health. The specific permit number for this study is: 1034/3887. The animals (typically 4 animals per cage; minimum floor space per animal– 100 cm2) were kept under controlled environmental conditions in the specific pathogen free animal facility with a 12L:12D cycle. Mice were housed in enriched IVC cages containing autoclaved bedding, nesting material and a smart home. They were provided with irradiated food and 0.22 μm filtered tap water. Mice were under visual inspection of their general health by animal facility dedicated staff and veterinary support is available if needed. This protocol is a terminal harvest, meaning no procedure was directly done on living conscious animals. They were humanely killed to harvest their spleen as a source for transgenic CTL. The mice were killed by lethal injection of 150 mg/kg of pentobarbital intraperitoneally and the spleen was resected after verification of the animal’s death. The lethal dose of pentobarbital prevent any suffering of the animal since with this procedure the animals die under profound anesthesia. CTLs were isolated from splenocytes. Mouse spleen was filtered through a 70 μm gauge strainer (Becton Dickinson) and incubated in red blood cell lysis buffer (RBC) (8.26 g ammonium chloride (NH_4_CI), 1 g potassium bicarbonate (KHCO_3_), 0.037 g EDTA dissolved in one liter of water) at 37°C for 10 minutes. Sample was spun for 5 minutes, at 315 G, and pellet was re-suspended in 1 ml of complete medium with 10 μg/ml OVA peptide (OVA 257–264: SIINFEKL) or human gp100 peptide (KVPRNQDWL) (Polypeptide group, USA) for 1 hour at 37°C. Cells were washed and maintained in RPMI 1640 medium. Every 48 hours, 50 U/ml of human interleukin-2 (Roche) were added. Cytotoxic T cells were used at day 6 and 9 of culture.

### Cell phenotyping

Surface expression of CD80, CD86, CD54, and MHC class I antigens were detected with anti-human or mouse CD86, CD80 (B7-1), MHC Class-I, and anti-human MICA/B from BD biosciences.

### FACS based CTL killing assay

Target cells were labeled with CFSE, and plated in a 96 U-bottom well plate with YT-Indy NK cells at various E:T ratio in a final volume of 100 μl. Cells were then stained with Draq7 (BioStatus Limited) according to the manufacture procedure then harvested and analysed by flow cytometry. The percentage specific lysis was calculated with the following formula:

(%of NK induced killing(CFSE,Draq7double positive in the presence of NK cells)−(%of spontaneous cell death(CFSE,Draq7double positive in the absence of NK cells)

### Calcein release cytotoxicity assay

Peptide loaded 10^6^ target cells were loaded with 1 μM Calcein AM for 15 minutes at 37°C in one ml final volume and then washed three times with HBSS. 1x10^4^ target cells per well (triplicate) in 0.1 ml medium (HBSS containing 1,55 mM CaCl_2_, 17,5 mM glucose, 10 mM Hepes, pH 7.4, 5% FBS and 5% supplement mixture) were incubated or not (control) with same volume of CTL at various E:T cell ratios in 96 well V-bottom plate. The target cells were leaded with OVA peptide or with human gp100 when using OT-1 or PMEL CTL respectively. Plates were spun at 85 g for one minute and incubated 4 hours at 37°C. After incubation, plates were spun for 5 minutes at 390 g and 80 μl/well of supernatants were transferred to a clear bottom black plate to read fluorescence using a 490/520 nm excitation/emission filter at SpectraMax, Paradigm Microplate Reader (Molecular device Sunnyvale, CA, USA).

$$\%\text{specific release}=100\frac{\mathrm{release}-\mathrm{spontaneous release}}{\text{max}\;\mathrm{release}-\mathrm{spontaneous release}}$$

### Two-color Calcein Release cytotoxicity assay

Two-color assay was used to compare the killing of two different target cell types simultaneously by the same effector cells. Target cells (10^6^ cells/ml) were loaded with 1μM calcein-AM while the second type of target cells were loaded with 1μM calcein-Red Orange (Invitrogen) for 15 minutes at 37°C. After three washes, target cells from both populations were mixed at a 1:1 ratio in the same well keeping the total target cell number 10^4^ in 0.1ml. Mixed target cells were incubated with 0.1 ml CTL at various E:T cell ratios. After 4 hours incubation, data were acquired by reading the fluorescence at 544/590 nm excitation/emission for the calcein red Orange labeled targets and 490/520 nm for the calcein AM labeled targets. % Lysis was calculated as mentioned before. Measurements of each fluorescence in the supernatant give the % of killing of each target cell type. For a given cell line it is expected that % killing obtained with calcein-AM assay = % killing obtained with calcein-RO assay. However since the calcein-RO is less effectively released than the calcein-AM, the specific lysis obtained from the calcein-RO must be adjusted with a correction factor (CF). For each target at each E:T ratio CF = % killing with calcein-AM assay—% killing with calcein-RO assay, obtained from single color assay for each calcein. After adjustment of calcein-RO data with the CF, the two calceins can be precisely compared. Any difference will reflect the actual difference between the target cell types. The dye swap experiments were performed to validate the results.

### Quantitative real-time reverse transcription polymerase chain reaction (qRT-PCR)

Total RNA were isolated from U251, hNS, GL261 and mNS using Trizol (Invitrogen) according to the manufacturer's instructions. RNA was reverse-transcribed using promega reverse transcription kit according to manufacture procedure. PCR was performed using SYBR Green PCR Master Mix on a Step One Plus Real-Time PCR system (Applied Biosystems) with a comparative CT (ΔΔCT) method. The values of the relative expression levels of individual genes are normalized to those of β-actin. qPCR primers list for GL261 and mNS were designed by IDT online oligonucleotide design application. (www.idtdna.com/pages/scitools)

B ACTIN FW: catcactattggcaacg ag

B ACTIN RV: tggcatag aggtctttacg

MELK FW: gggcaacaaggactaccatc

MELK RV: tgggagagagccacttagga

CD90 FW: catcagcgtcgctctcct

CD90 RV: ctgaactcatgctggatgga

CD44 FW: ccagtgacccctgctaaaac

CD44 RV: cctggagtccttggatgagt

CXCR4 FW: tcagtggctgacctcctctt

CXCR4 RV: cttggcctttgactgttggt

PTCH FW: ctcaggcaatacgaagcaca

PTCH RV: gacaaggagccagagtccag

CD 133 FW: tgaaaagttgctctgcgaacc

CD 133 RV: tctcaagctgaaaagcagca

MSI1 FW: gaggactcagttggcagacc

MSI1 RV: ctgtgctcttcgaggaaagg

Primers for U251 and hNS:

BETA-ACTIN FW: ttctacaatgagctgcgtgtg

BETA-ACTIN RV: ggggtgttgaaggtctcaaa

MELK FW: cttggatcagaggcagatgtttggag

MELK RV: gttgtaatcttgcatgatccagg

CD90 FW: cgctctcctgctaacagtctt

CD90 RV: caggctgaactcgtactgga

CD44 FW: agaaggtgtgggcagaagaa

CD44 RV: aaatgcaccatttcctgaga

PTCH FW: cgcctatgcctgtctaaccatgc

PTCH RV: aaatggcaaaacctgagttg

Primers for the cytotoxic molecules

GA

FW: cactcaagaccgtatatggctc

RV: agtgagccccaagaatgaac

GB

FW: cctccaggacaaaggcag

RV: cagtcagcacaaagtcctctc

GC

FW: gtgggagactcaaagatcaagg

RV: tgtgaagacttgtggagctg

GD

FW: agatgactttgtgctgacgg

RV: gaatgtcttttgccacaggg

GE

FW: gattctcctgaccctacttctg

RV: gcctccacagtatctcctattac

GF

FW: agatttgtgctggagaccc

RV: caaccttagtgaagactcctgg

GG

FW: gattctcctgaccctacttctg

RV: cttcccttcgatatccacagac

GK

FW: ggagatgccagaggtcaaaag

RV: tgccacatttatagccctgag

GM

FW: atcggaagtgggtattgacag

RV: gttgtagccagggtgtttaatg

GN

FW: agagatttgtgctggagacc

RV: cacaaccttagtgaagactcctg

P

FW: cagtagagtgtcgcatgtacag

RV: gagatgagcctgtggtaagc

### P and GB treatment

Perforin (P) was purified from the rat RNK-16 cells and GB from the human YT-Indy NK cells. GB was first added to cells (5x10^4^) suspended in 30 μl cell buffer (HBSS with 10 mM HEPES, pH 7.4, 0.4% BSA, 4 mM CaCl_2_) and immediately thereafter an equal volume of P in HBSS with 10 mM HEPES, pH 7.4, was added. The P concentration was chosen as the sublytic concentration that lyses 5%–15% of cells. Cells were incubated at 37°C for one hour before staining for annexin V and propidium iodine (PI). Samples were analyzed with an Accuri C6 flow cytometer (BD Biosciences).

### Statistical Analysis

Data represent mean +/- SD of indicated number of independent experiments. Statistical significance has been calculated by one-tailed Student t-test between samples and P values are indicated in the legends.

## Results

### Glioma stemlike cells are more sensitive to cytotoxic immune cells

**Fig 1:**
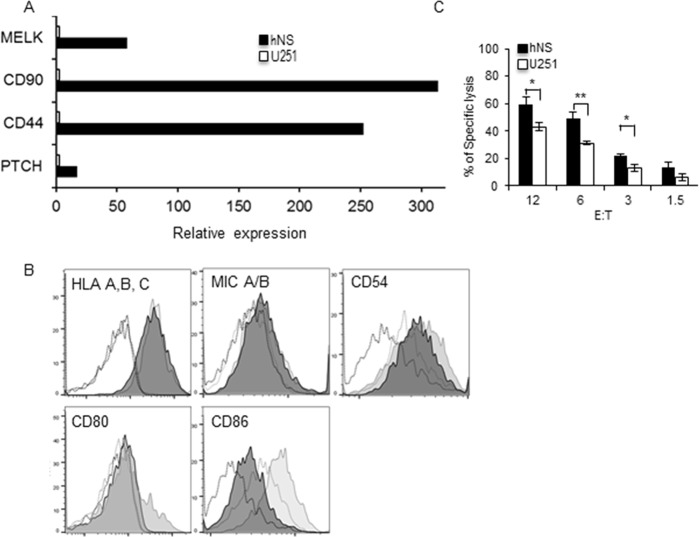
Human Glioma stemlike cells are better killed by YT-Indy NK cells. (A) Messenger RNA expression of the stem cell markers MELK, CD90, CD44 and PTCH, for U251 differentiated glioma cells and hNS cells assessed by qRT-PCR. (B) Phenotyping of U251 and hNS cells for the surface expression of MHC class I, MICA/B, CD54, CD80, CD86. Filled dark and light grey histograms are for hNS and U251 respectively, open dark and light grey histograms are the corresponding controls. (C) U251 and hNS were also used as target YT-Indy in a 4 hour calcein AM release assay. All data are representative of 3 independent experiments. Bar graphs represent mean +/- SD of three independent experiments. P value * p ≤ 0.05, ** p ≤ 0.01, one-sided t-test.

In order to test which of GSC and the GDC are more sensitive to cytotoxic immune cells we first used U251 human glioma differentiated cells (GDC) and their neurosphere (hNS) GSC counterpart to investigate their relative susceptibility to natural killer cells. We first confirmed that hNS GSC have a higher expression level of the stem cell markers MELK, CD90, CD44 and PTCH [19, 29–32] than U251 GDC cells (Fig 1A). Moreover, there was no significant difference in the expression of MHC class I, MICA/B NK cell activator ligand, the costimulatory molecules CD80/B7.1, and CD54 adhesion molecules while the co-stimulatory receptor CD86/B7.2, was more expressed by the U251 GDC (Fig 1B). Next using a FACS based approach we tested the differential sensitivity of U251 GDC and hNS GSC to YT-Indy killer cells. U251 and hNS were first labeled with CFSE before incubation with various ratio of effector cells and cell death assessed four hours later by Draq7 staining and FACS analysis of the CFSE-positive cells (Fig 1A in S1 File). At all the effector: target (E:T) ratio tested, hNS GSC were significantly better killed by the YT-Indy NK effector cells (Fig 1A and B in S1 File). This results was also true when using U251 GDC and hNS as targets of YT-Indy in a four hour calcein release experiment (Fig 1C). Interestingly in the calcein based assay U251, killing was slightly higher than with the FACS based assay. This could be due to the different sensitivity of the two assays.

**Fig 2:**
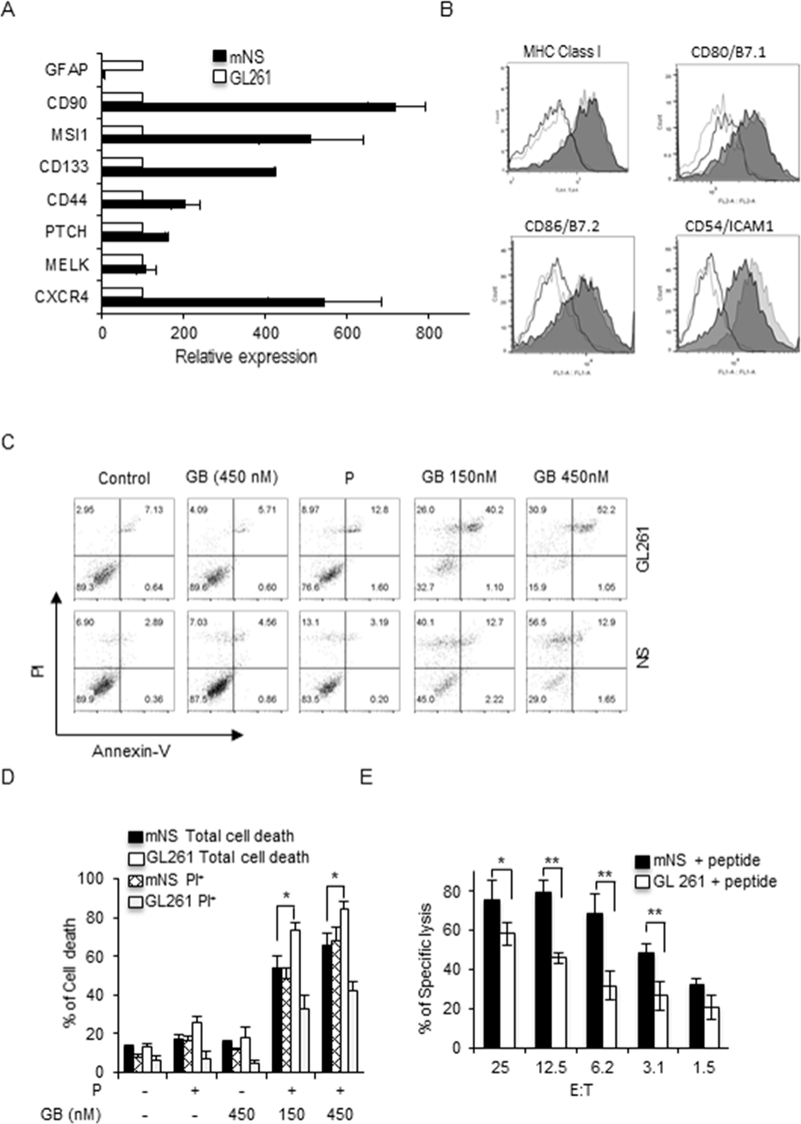
Glioma stemlike cells are better killed by CTL. (A) Messenger RNA expression of the stem cell markers CD90, MSl1, CD133, CD44, PTCH, MELK, CXCR4 and differentiation marker GFAP for GL261 and mNS cells assessed by qRT-PCR. (B) Phenotyping of GL261 and mNS cells for the surface expression of MHC class I, CD54, CD80, CD86. Filled dark and light grey histograms are for mNS and GL261 respectively, open dark and light grey histograms are the corresponding controls. (C) GL261 and mNS were treated with a sublytic dose of perforin (P) and granzyme B (GB) for one hour and cell death monitored by annexin V-PI staining. (D) Same as in C mean +/-SD of three independent experiment. (E) GL261 and mNS were pulsed or not with peptide and used as target cells for day 6 PMEL CTL in a 4 hour calcein AM release assay. All data are representative of 3 independent experiments. Bar graphs represent mean +/- SD of three independent experiments. P value * p ≤ 0.05, ** p ≤ 0.01, one-sided t-test.

We also used the mouse GL261 GDC and their neurosphere (mNS) GSC counterpart to investigate their respective susceptibility to CTL killing. As before we confirmed that mNS GSC have a higher expression level of the stem cell markers CXCR4, MELK, PTCH, CD44, CD133, MSl1 and CD90 [19, 29–32], while GL261 GDC expressed higher level of the differentiated glioma marker glial fibrillary acidic protein (GFAP) (Fig 2A). Here also there was no significant difference in the expression of MHC class I and of the costimulatory molecules CD80/B7.1 and CD86/B7.2, while the adhesion molecule CD54/ICAM1 was slightly more expressed on GL261 GDC cells than on mNS GSC (Fig 2B). Since granzyme B (GB) is one of the most potent of the 10 mouse granzymes involved in the cytotoxic granule pathway [33], we then tested the susceptibility of GL261 and mNS cells to human GB and perforin (P) induced cell death. GB and P treatment induced a dose-dependent cell death in both GL261 and mNS. Interestingly, in the loading experiments used to mimic CTL attack, GL261 cells were more sensitive to GB-mediated cell death than mNS cells as seen by annexin V-PI staining (Fig 2C and 2D). In fact, mNS cells died exclusively in a necrotic-like manner since all the dead mNS cells were single positive for PI. GL261 dead cells encompassed necrotic-like PI single positive, but also apoptotic annexin single positive and annexin-PI double positive cells (Fig 2C and 2D). These results indicate that GDC and GSC are sensitive to GB although their death seems to involve different pathways. We next used these glioma cells as target for PMEL or OT-1 CTL isolated from PMEL and OT-1 mice (Charles River) bred in house and used between 6–12 weeks of age according to approved animal protocols. PMEL CTL express a rearranged T cell receptor (TCR) transgene specific for peptide 25–33 of mouse gp100 presented by H2-D^b^ MHC class I molecules [34]. These CTL were isolated from splenocytes and used at day 6 and day 9. Based on the expression profile of the granzymes and perforin cytotoxic molecules, we considered day 6 CTL less mature terminal effectors than day 9 CTL (Fig 2A and B in in S1 File) [35]. Day 6 PMEL CTL efficiently killed GL261 and mNS cells in a peptide-dependent and dose-dependent manner with mNS consistently better killed than GL261 at all the E:T ratios (Fig 2E) in agreement with previous reports [20–26]. We did not observe any significant difference in adherent and non-adherent GL261 killing (Fig 2C in S1 File). Lastly both GL261 and mNS cells slightly altered the viability of the day 6 CTL except at the lowest E:T ratio where the CTL faced an excess of cancer cells (Fig 2D in S1 File). Taken together these results confirmed that both human and mouse GSC and GDC are efficiently killed by NK cells and CTL with GSC being even more sensitive than GDC.

### GSC potentiate PMEL CTL killing of GDC

**Fig 3:**
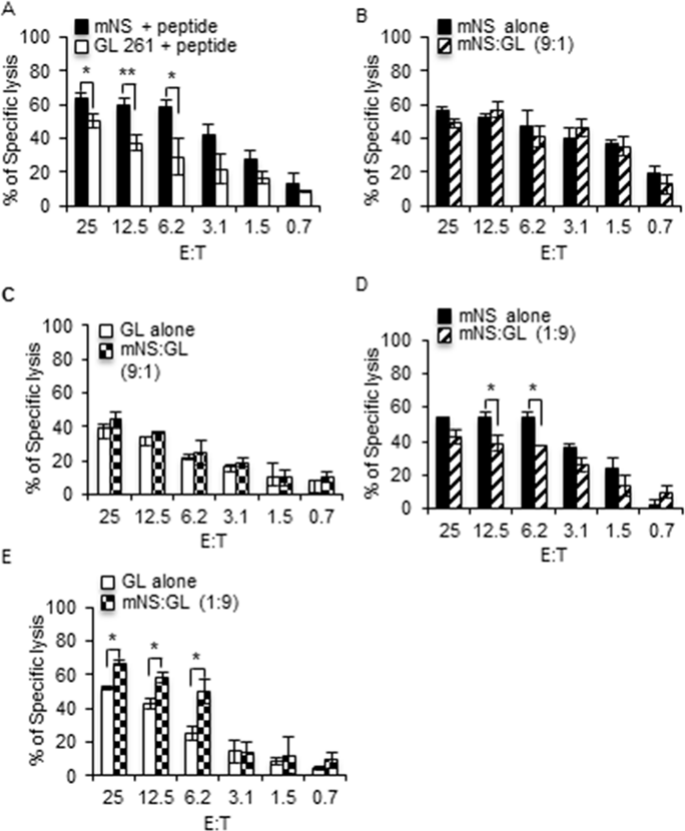
GSC enhance the killing of GDC by day 6 PMEL CTL. (A) GL261 and mNS were loaded with calcein AM and calcein Red Orange respectively, then pulsed with peptide and mixed in 1:1 ratio before simultaneous incubation with day 6 PMEL CTL in a dual color cytotoxicity assay. The cytotoxicity toward each target is followed by measuring the release of the respective calcein in the supernatant. (B) Peptide-pulsed mNS cells alone were used as target for day 6 PMEL CTL in a classical calcein AM release assay (mNS alone), while on the other side peptide-pulsed mNS were mixed in 9:1 ratio with peptide-pulsed GL261 (mNS:GL 9:1) and incubated simultaneously with day 6 PMEL CTL in a dual color cytotoxicity assay. (C) The killing of GL261 facing CTL alone were compared with the killing of GL261 in a mNS:GL ratio of 9:1 obtained from B. (D) Same as in B for an mNS:GL ration of 1:9. (E) Same as in C for mNS:GL ration of 1:9. All bar graphs are mean +/- SD of three independent experiments. P value * p ≤ 0.05, ** p ≤ 0.01, one-sided t-test.

**Fig 4:**
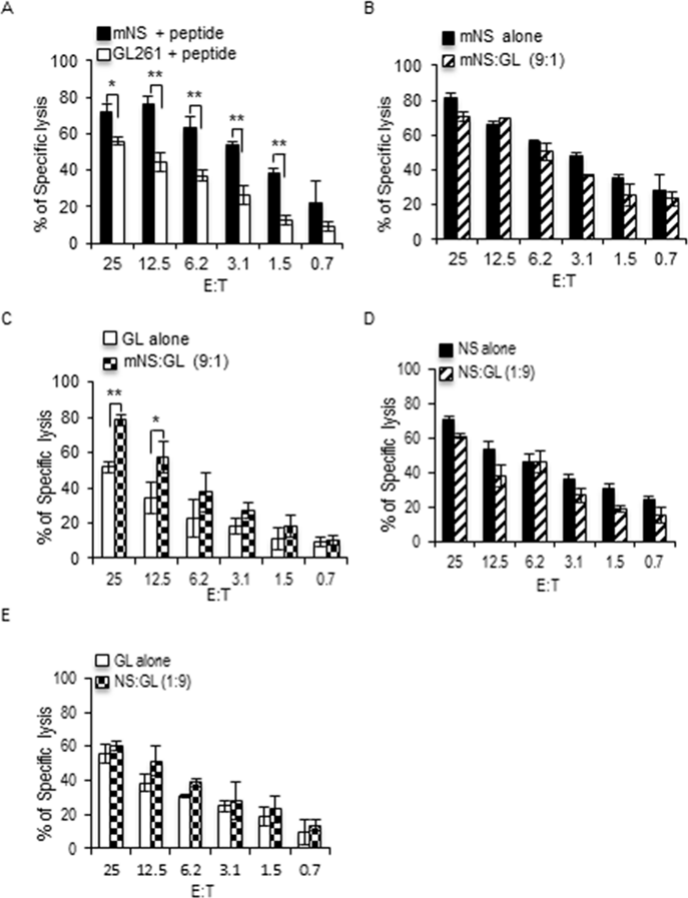
GSC enhance the killing of GDC by day 9 PMEL CTL. (A) GL261 and mNS were loaded with calcein AM and calcein Red Orange respectively, then pulsed with peptide and mixed in 1:1 ratio before simultaneous incubation with day 9 PMEL CTL in a dual color cytotoxicity assay. The cytotoxicity toward each target is followed by measuring the release of the respective calcein in the supernatant. (B) Peptide-pulsed mNS cells alone were used as target for day 9 PMEL CTL in a classical calcein AM release assay (mNS alone), while on the other side peptide-pulsed mNS were mixed in 9:1 ratio with peptide-pulsed GL261 (mNS:GL 9:1) and incubated simultaneously with day 9 PMEL CTL in a dual color cytotoxicity assay. (C) The killing of GL261 facing CTL alone compared with the killing of GL261 in mNS:GL ratio of 9:1 obtained from B. (D) Same as in B for an mNS:GL ratio of 1:9. (E) Same as in C for mNS:GL ratio of 1:9. All bar graphs are mean +/- SD of three independent experiments. P value * p ≤ 0.05, ** p ≤ 0.01, one-sided t-test.

Next we aimed at testing the influence of GSC on GDC killing and *vice versa*. To address this question, we took advantage of the different emissions of fluorescent calcein dyes and developed a two-color cytotoxicity assay. GL261 loaded with calcein AM were mixed with mNS loaded with calcein red orange at different GL261:mNS ratio and then incubated with PMEL CTL for four hours. Cytotoxicity toward each type of target cells was assessed by quantifying the release in the supernatant of the corresponding calcein. As show in Fig 3A, using this novel assay, we confirmed that mNS were better killed than GL261 by day 6 PMEL CTL at all the E:T ratios (Fig 3A). Similar results were obtained in the dye swap experiment where this time, mNS were loaded with calcein AM and GL261 with calcein red orange (Data not shown). We then compared the killing of mNS facing the CTL alone or in the presence of a minority (mNS:GL = 9:1) or an excess (mNS:GL = 1:9) of GL261 GDC cells. In this case mNS facing CTL alone were tested in a classical single color calcein release assay, while mNS facing CTL in the presence of GL261 were done in dual color assay to monitor both mNS and GL261 killing. Day 6 PMEL CTL killed mNS to a similar extent whether alone or in the presence of a small number of GL261 (Fig 3B). Unexpectedly, this small number of GL261 was killed to a similar extent as the same number of GL261 alone (Fig 3C) indicating that dilution of GL261 in the large excess of mNS cells did not protect them from day 6 PMEL CTL killing. We next tested the effect of an excess of GL261 on the killing of mNS cells. Mouse NS cells were slightly less killed when diluted in an excess of GL261 (Fig 3D), while unexpectedly, this small number of mNS potentiates the killing of this large number of GL261 at the highest E:T ratio (Fig 3E). We then repeated the same experiment using more mature terminal effector day 9 PMEL CTL. In this context, mNS cells were still better killed than GL261 (Fig 4A). As previously, a small number of GL261 did not affect the killing of mNS (Fig 4B). However, this small number of GL261 cells were better killed when facing CTL in an excess of mNS cells compared to GL261 alone (Fig 4C). In the reciprocal setting, an excess of GL261 still mildly affected the killing of mNS cells (Fig 4D), but this time, the small number of mNS barely affected the cytotoxicity toward GL261 (Fig 4E). Note that the viability of the more mature PMEL CTL were more affected by both type of cancer cells (Fig 3 in S1 File) compared to that of the less mature day 6 CTL (Fig 2C in S1 File). Taken together, although mild, excess of GL261 has a protective effect on the killing of mNS cells that seems to depend on the maturity of the PMEL CTL. However whether in excess or in minority, the presence of mNS cells seems to potentiate the killing of the GL261 GDC.

### GSC potentiate OT-1 CTL killing of GDC

**Fig 5:**
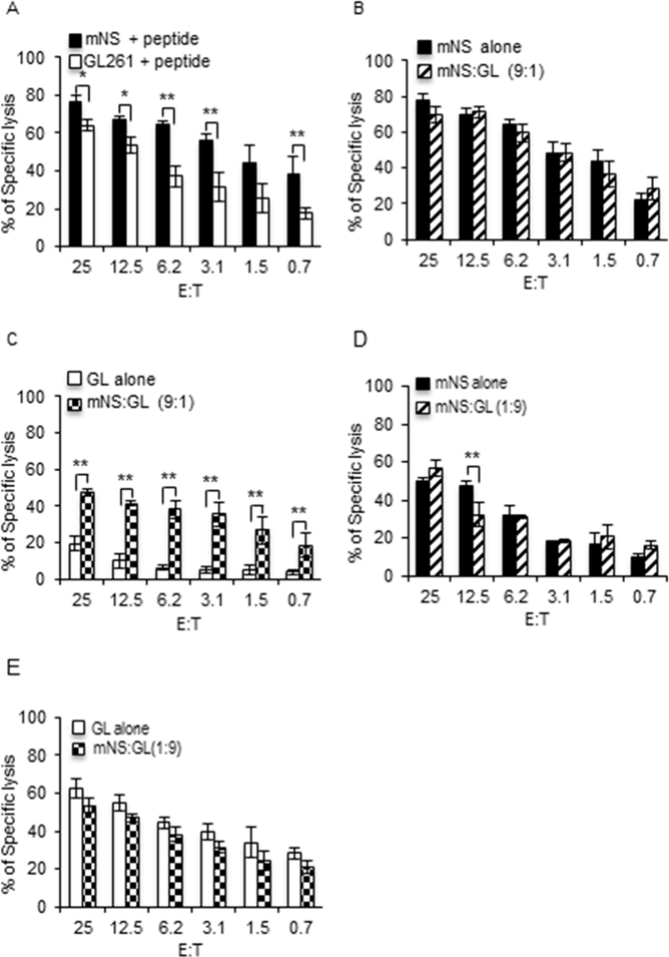
GSC enhance the killing of GDC by day 6 OT-1 CTL. (A) GL261 and mNS were loaded with calcein AM and calcein Red Orange respectively, then pulsed with peptide and mixed in 1:1 ratio before simultaneous incubation with day 6 OT-1 CTL in a dual color cytotoxicity assay. The cytotoxicity toward each target is followed by measuring the release of the respective calcein in the supernatant. (B) Peptide-pulsed mNS cells alone were used as targets for day 6 OT-1 CTL in a classical calcein AM release assay (mNS alone), while on the other side peptide-pulsed mNS were mixed in 9:1 ratio with peptide-pulsed GL261 (mNS:GL 9:1) and incubated simultaneously with day 6 OT-1 CTL in a dual color cytotoxicity assay. (C) The killing of GL261 facing CTL alone compared with the killing of GL261 in mNS:GL ratio of 9:1 obtained from B. (D) Same as in B for an mNS:GL ratio of 1:9. (E) Same as in C for mNS:GL ratio of 1:9. All bar graphs are mean +/- SD of three independent experiments. P value * p ≤ 0.05, ** p ≤ 0.01, one-sided t-test.

We next aimed to validate these results using the very potent OT-1 CTL expressing a transgenic TCR specific for chicken ovalbumin residues 257–264 in the context of H2K^b^ MHC I molecule [36]. Day 6 OT-1 CTL also better killed mNS than GL261 at all the E:T ratio tested (Fig 5A). While a small number of GL261 had no effect on day 6 OT-1 killing of mNS cells (Fig 5B), this small number of GL261 cells were far better killed when diluted in the excess of mNS than when facing the CTL alone (Fig 5C). In the reciprocal setting, excess of GL261 did not influence the killing of a small number of mNS cells (Fig 5D). Similarly, OT-1 CTL killing of GL261 was not affected by the number of mNS cells (Fig 5E). This was even more evident when using more day 9 OT-1 CTL. Mouse NS GSC were still better killed by day 9 OT-1 CTL (Fig 4A in S1 File) however, this was not affected by the presence of GL261, nor was the killing of GL261 affected by the presence of mNS cells (Fig 4B-E in S1 File). Overall, our results indicate that the presence of mNS GSC potentiates the killing of the GL261 GDC, while GL261 has a mild protective effect on mNS, both to an extent that is dependent on the maturity and the potency of the CTL involved.

## Discussion

Using U251 human and GL261 mouse glioma models and YT-Indy natural killer cells or PMEL and OT-1 CTL, two well characterized mouse killer lymphocyte models, we confirmed that both glioma differentiated cells (GDC) and neurosphere (NS) glioma stemlike cells (GSC) are effectively killed by natural killer cells and cytotoxic T lymphocytes. We also confirmed that NS GSC were even better killed than the GDC in both human and mouse glioma models at all the E: T ratio tested and this regardless of the affinity and maturity of the cytotoxic T lymphocytes used in these experiments, in line with previous reports [20–26]. The higher susceptibility of NS to cytotoxic effector cells was not due to a difference in the surface expression of MHC class I or costimulatory molecules CD80 and CD86, nor to a defect in their death machinery since both GL261 and mNS cells are sensitive to GB. Whether this observed higher susceptibility of mNS GSC to CTL can be generalized to all GSCs will require testing with additional pairs of GDC and GSC. Interestingly, following GB and P treatment, mNS cells died in a necrotic-like manner compared with their GL261 GDC counterpart. This could be the consequence of the anaerobic glycolysis metabolic shift observed in cancer stem cells [37] compared to the well documented aerobic glycolysis of differentiated cancer cells such as GL261 cells (data not showed) [38, 39]. This anaerobic glycolysis is less likely capable of providing the energy necessary for apoptosis shifting the death from apoptosis to necrosis-like [40]. Since GL261 were less efficiently killed by CTL, we initially expected that they would shield the mNS from the killer cells. We indeed observed such a protective effect although very mild and dependent on the level of maturity and how strongly the cytotoxic granzymes and perforin are expressed by the CTL used. This could be the result of a dilution of the mNS in the excess of differentiated glioma cells decreasing the probability for mNS to meet an effector cell. Such a ratio of GSC:GDC is most likely relevant to the *in vivo* tumor where glioma stemlike cells are expected to be in smaller number compared to the glioma differentiated cells. In this case our results suggest that during combination therapy, modalities aimed at depleting the vast majority of the tumor bulk would better synergize with immunotherapy if used first. This staging of the therapeutic intervention would be even more critical when dealing with low potency CTL, as might be expected for low-avidity T cells targeting shared self/tumor-associated antigens. Unexpectedly, we observed that the presence of mNS whether in excess or in small numbers increased the killing of GL261, with the most obvious effect observed with day 6 OT-1 CTL facing a small number of GL261 buried in an excess of mNS cells. This suggests a putative collateral damage associated with the massive loss of the mNS that are better killed by the CTL. This also further emphasizes that treating glioma with conventional therapies, expected to be more efficient at killing the differentiated cancer cells, should provide the optimal conditions for subsequent immunotherapy. In such a setting, the GSC:GDC ratio should be in favor of the GSC, exposing them to the CTL and enhancing the eradication of the remaining differentiated cancer cells which escaped the initial treatment. Therefore, understanding the mechanism supporting the higher susceptibility of the glioma stem cells to CTL than the glioma differentiated cells would be of tremendous importance in order to fine tailor next generation immunotherapy.

## Supporting Information

S1 File GSC potentiate the killing of GDC Figs 1–4. (PDF)
