# Supplementary material for: Glioma Stemlike Cells Enhance the Killing of Glioma Differentiated Cells by Cytotoxic Lymphocytes
Source: PLoS One. 2016 Apr 13;11(4):e0153433. doi: 10.1371/journal.pone.0153433 (PMC4830556; doi:10.1371/journal.pone.0153433)
Supplement: S1 File — (PDF) [file pone.0153433.s001.pdf]

Glioma stemlike cells enhance the killing of glioma differentiated cells by cytotoxic T lymphocytes. Esen Yonca Bassoy[1], Valentina Chiusolo[1], Guillaume Jacquemin[1], Cristina Riccadonna[4], Paul R. Walker[4] and Denis Martinvalet*[1].

Supplemental Figures and Legends:

Bassoy et al Figure S1

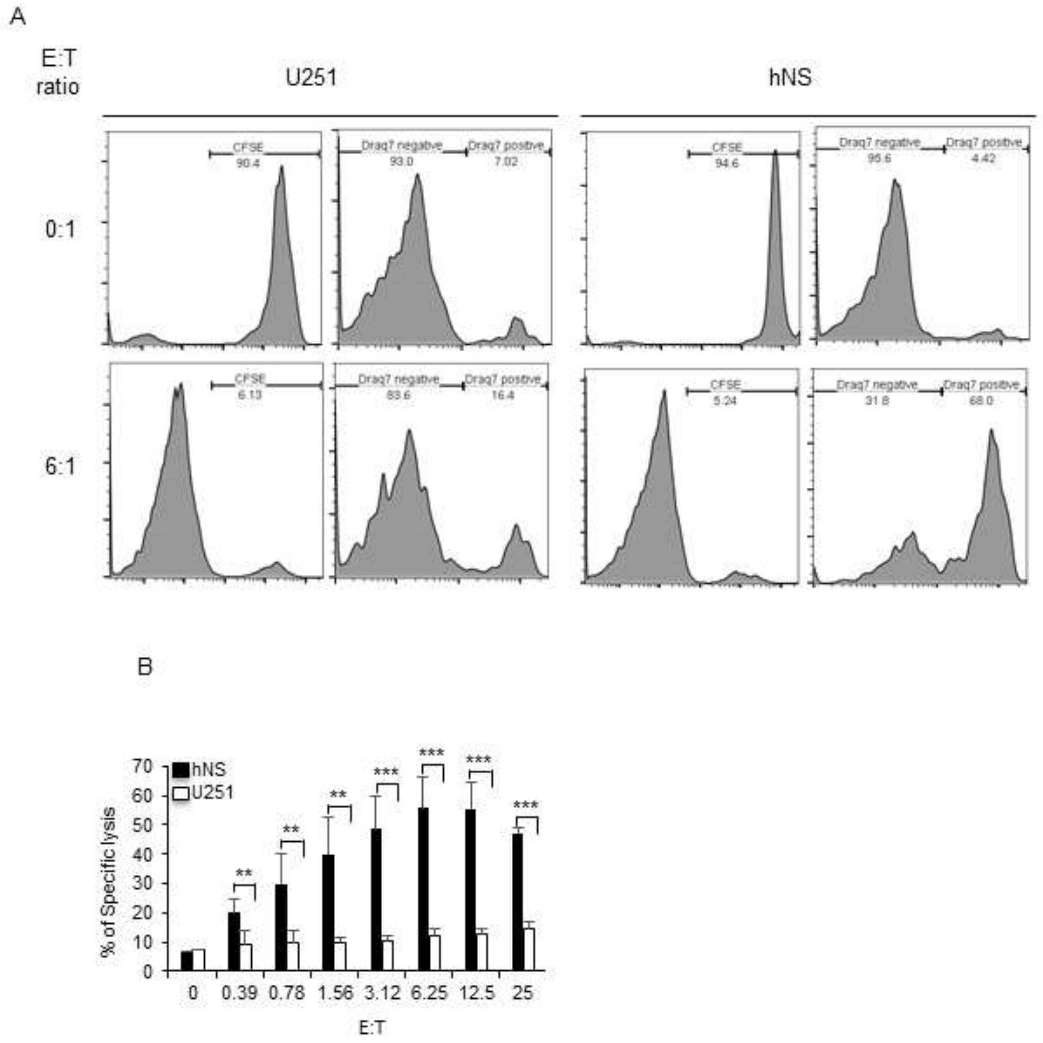

Figure S1: Human NS are better killed than U251 glioma cell by YT-Indy NK cells. (A) U251 and hNS were labeled with CFSE before incubation separately with YT-Indy NK cells a different E:T ratio for 4 hours. Cell death was monitored by Draq7 staining and FACS

analysis gating on the CFSE positive cells. (B) Same as in C mean +/-SD of three independent experiment.

Bassoy et al Figure S2

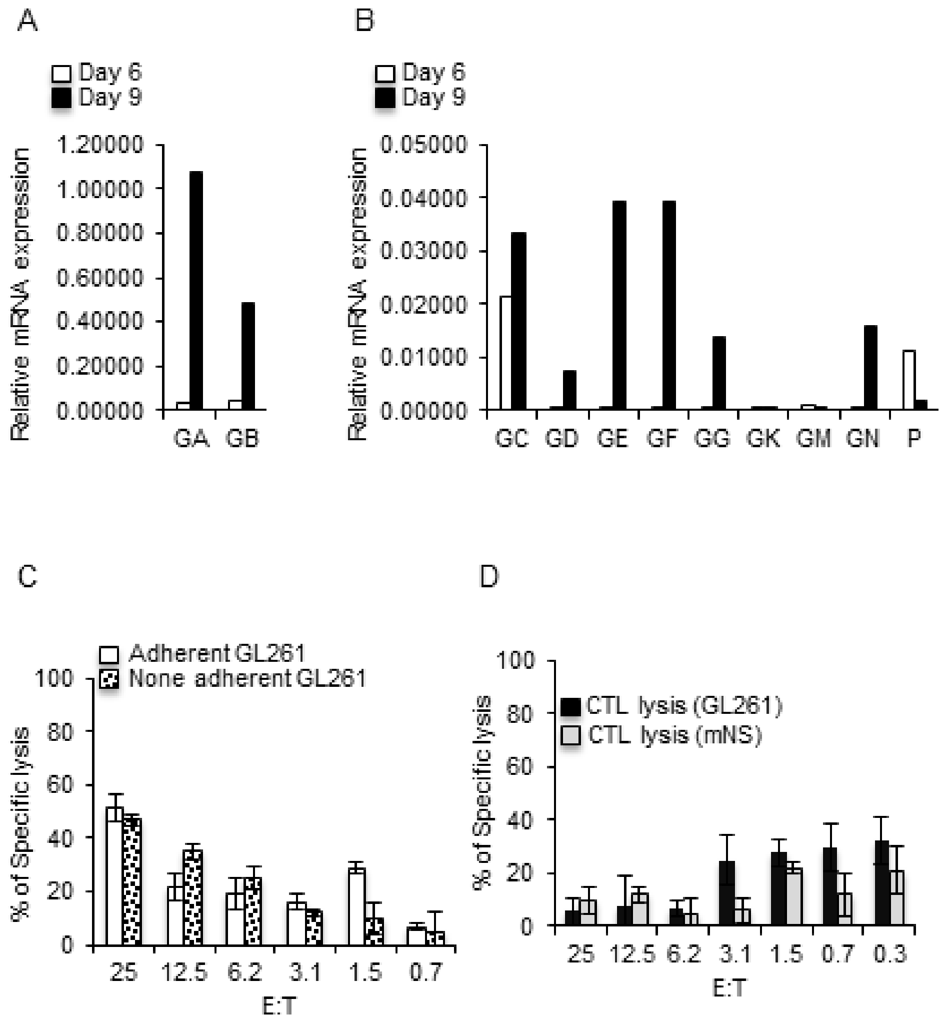

Suppl. Fig 2: CTL kill adherent and none adherent GL261 similarly. (A and B) PMEL CTL were isolated from splenocytes stimulated *in vitro* with gp100 peptide. 50 U/ml IL2 were added every 48 hours. A day 6 and 9 mRNA were isolated from the CTL to analyze the expression of granzymes (A, B, C, D, E, F, G, K, M and N) and perforin (P). (C) Adherent and none adherent GL261 cells were used as targets for day 6 PMEL CTL in single classical calcein AM release assay. (D) Day 6 PMEL CTL were loaded with calcein AM and incubated

with either GL261 or NS cells and the CTL death was followed in a classical calcein AM release assay. Bar graphs are mean +/- SD of three independent experiments.

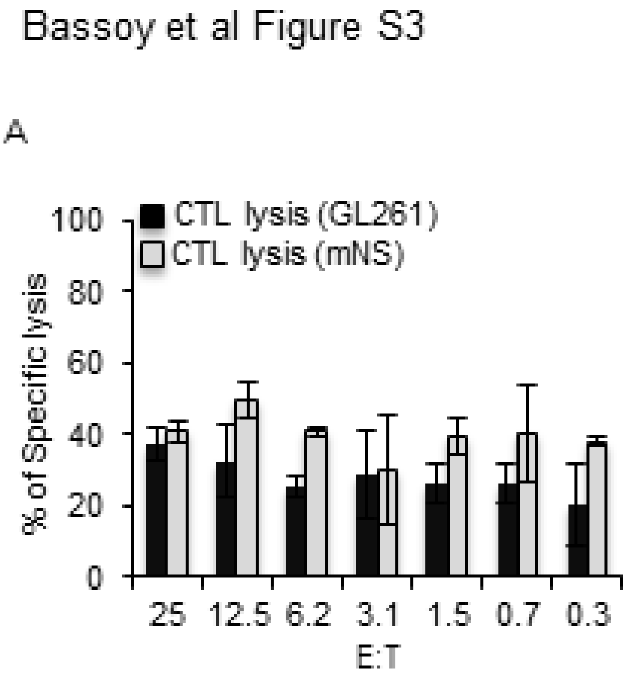

Figure S3: Cancer cells alter CTL viability. Day 9 PMEL CTL were loaded with calcein AM and incubated with either GL261 or mNS cells and the CTL death was followed in a classical calcein AM release assay. Bar graphs are mean +/- SD of three independent experiments.

Bassoy et al S4

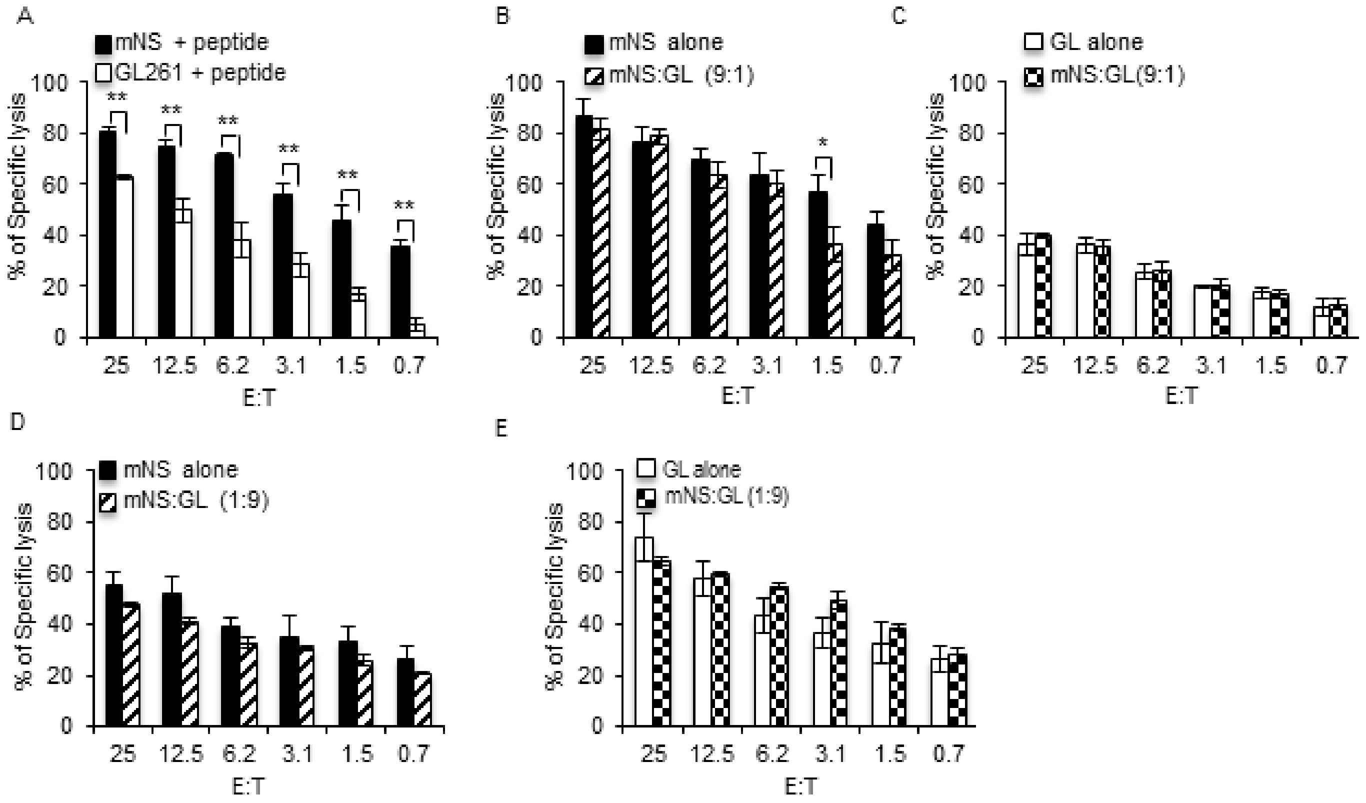

Figure S4: Glioma stemlike cells are better killed by day 9 OT-1 CTL. (A) GL261 and mNS were loaded with calcein AM and calcein Red Orange respectively, then pulsed with peptide and mixed in 1:1 ration before simultaneous incubation with day 9 OT-1 CTL in a dual color cytotoxicity assay. The cytotoxicity toward each target is followed by measuring the release of the respective calcein in the supernatant. (B) Peptide-pulsed mNS cells alone were used as targets for day 9 OT-1 CTL in a classical calcein AM release assay (mNS alone) while on the other side peptide-pulsed mNS were mix in 9:1 ratio with peptide-pulsed GL261 (mNS:GL 9:1) and incubated simultaneously with day 9 OT-1 CTL in a dual color cytotoxicity assay. (C) The killing of GL261 facing CTL alone compared with the killing of GL261 in a mNS:GL ratio of 9:1 obtained from B. (D) Same as in B for an mNS:GL ratio of 1:9. (E) Same as in C

for mNS:GL ratio of 1:9. All bar graphs are mean +/- SD of three independent experiments. P value * $p \leq 0.05$, ** $p \leq 0.01$, one-sided t-test.
